# Letter to the Editor: Evaluating the efficacy of OLIF combined with pedicle screw internal fixation for lumbar spinal stenosis

**DOI:** 10.1186/s13018-023-04260-z

**Published:** 2023-11-16

**Authors:** Fatemeh Ranjbari, Ehsan Alimohammadi

**Affiliations:** 1 Clinical Research Development Center, Imam Reza Hospital, Kermanshah University of Medical Sciences, Kermanshah, Iran; 2 Department of Neurosurgery, Imam Reza Hospital, Kermanshah University of Medical Sciences, Kermanshah, Iran

Dear Editor,

We are writing to discuss the recent article titled “Efficacy of OLIF combined with pedicle screw internal fixation for lumbar spinal stenosis on spinal canal changes before and after surgery” by Xu et al. [1] published in the Journal of Orthopaedic Surgery and Research. This study aimed to evaluate the effectiveness of OLIF (oblique lateral interbody fusion) combined with pedicle screw internal fixation in treating lumbar spinal stenosis by assessing the changes in the spinal canal before and after surgery.

The authors conducted a retrospective study involving sixteen patients who underwent a combination of single-segment OLIF and pedicle screw internal fixation for the treatment of lumbar spinal stenosis. They compared the patients’ pre- and post-operative data and observed various parameters such as intraoperative bleeding, duration of surgery, visual analogue score (VAS), Oswestry Disability Index (ODI), disc height (DH), cross-sectional area of the vertebral canal (CSAVC), cross-sectional area of the dural sac (CSADS), cross-sectional area of the intervertebral foramen (CSAIF), spinal canal volume (SCV), spinal canal volume expansion rate, lumbar lordosis, and sagittal vertical axis.

The results of the study demonstrated that OLIF combined with pedicle screw internal fixation effectively restored disc height and increased the cross-sectional area of the spinal canal. This technique also had an indirect decompression effect. The intraoperative bleeding and duration of surgery were within acceptable ranges. Furthermore, the VAS and ODI scores significantly improved after surgery, indicating a reduction in pain and improvement in functional disability. The CSAVC, CSADS, CSAIF, SCV, and spinal canal volume expansion rate were all increased postoperatively. Additionally, there was improvement in lumbar lordosis and sagittal vertical axis. The follow-up conducted at 1 year after surgery showed that the parameter values remained statistically significant compared to the preoperative values.

While the study provides valuable insights, it is important to acknowledge certain limitations.

1. *Heterogeneous patient population* The study population in their research was heterogeneous, with different lumbar levels and pathologies. This can make it challenging to draw definitive conclusions about the effectiveness of the procedure for specific subgroups of patients.
2. *Small sample sizes* Small sample sizes can limit the generalizability of the findings. Larger studies with more participants are needed to provide more robust evidence.
3. *Short follow-up duration* Longer-term follow-up is necessary to assess the durability and long-term outcomes of the procedure.
4. *Lack of comparison groups* There was no direct comparison between OLIF combined with pedicle screw internal fixation and other treatment approaches or control groups. Comparative studies are essential to determine the relative efficacy and safety of this procedure compared to alternative treatments.
5. *Selection bias* There may have been selection bias in their patient cohorts, which can affect the generalizability of the results.

In a retrospective study, He et al. [2] compared standalone oblique lateral interbody fusion (OLIF) versus OLIF combined with posterior bilateral percutaneous pedicle screw fixation (OLIF combined) for the treatment of lumbar spondylolisthesis. Here are some key findings from the study:

1. *Clinical outcomes* The study found that the standalone OLIF group achieved better clinical outcomes in the early postoperative term (1 week and 3 months), but the differences disappeared by 2 years. This could be because standalone OLIF does not invade the muscle groups on both sides of the spine and does not cause intra-muscular hematoma, leading to better early recovery.
2. *Cage subsidence* The rate of cage subsidence was 15.6% in the standalone OLIF group and 7.3% in the combined OLIF group. The study suggests that cage subsidence may occur after OLIF combined with pedicle screw fixation as well, but theoretically, pedicle screw fixation could add more protection. Patients who are osteoporotic or obese might be better undergoing combined OLIF with pedicle screw fixation.
3. *Fusion rate* The study found no difference in fusion rate between standalone OLIF and OLIF combined with pedicle screw fixation within 2 years of follow-up. Despite being more unstable compared to OLIF combined with screw implantation, standalone OLIF with intact bilateral muscles could help stabilize the spine, resulting in a similar fusion rate.
4. *Cage subsidence and adjacent vertebral fractures* The study suggests that cage subsidence and adjacent vertebral fractures could be mutually causal. Endplate damage during surgery can cause trabecular bone fracture in the vertebral body, leading to cage subsidence. The study emphasizes the importance of careful surgical technique to minimize endplate damage.

In another study, Liu et al. [3] provided valuable insights into the outcomes of two surgical approaches, oblique lateral interbody fusion (OLIF) and minimally invasive transforaminal lumbar interbody fusion (MISTLIF), for the treatment of mild-to-moderate symptomatic degenerative lumbar spinal stenosis (DLSS).

The authors conducted a retrospective cohort study comparing OLIF and MISTLIF in patients with L4/5 DLSS. They evaluated various perioperative factors such as operative time, blood loss, hospital stay, cost, and complications. Additionally, they assessed radiographic changes and clinical outcomes, including visual analog scale (VAS) scores, Oswestry Disability Index (ODI), and Hospital Anxiety and Depression Scale (HADS).

The findings of this study are intriguing. The OLIF group demonstrated advantages in terms of shorter bedtime, reduced postoperative hospital stays, and lower intraoperative and postoperative blood loss compared to the MISTLIF group. However, the OLIF group required more intraoperative fluoroscopy, had a longer operative time, and incurred higher costs. Importantly, the complication rates were similar between the two groups.

Radiographic measurements revealed that OLIF resulted in significant increases in posterior intervertebral space height (PISH), intervertebral space foramen height (IFH), and intervertebral foramen area (IFA) compared to MISTLIF. Both surgical approaches led to a significant increase in the area of the spinal canal (ASC), but the ASC increase was more pronounced in the MISTLIF group.

Regarding clinical outcomes, the OLIF group exhibited significantly lower lumbar VAS scores at 1 month and 6 months post-operation. There were no significant differences in lower extremity VAS and ODI scores between the two groups. Notably, the OLIF group showed a significant decrease in HADS scores on postoperative day 3 and prior to hospital discharge, indicating a reduction in anxiety levels compared to the MISTLIF group.

These findings suggest that OLIF may offer several advantages over MISTLIF in the treatment of mild-to-moderate symptomatic DLSS. OLIF appears to result in less surgical invasion, lower incidence of postoperative low back pain, faster postoperative recovery, and reduced anxiety levels. However, it is important to consider the higher cost and longer operative time associated with OLIF.

In conclusion, the study suggests that OLIF combined with pedicle screw internal fixation is an effective treatment option for lumbar spinal stenosis. It not only restores disc height and increases the cross-sectional area of the vertebral canal but also provides valuable information for evaluating the efficacy and functional recovery of the lumbar spine in patients with lumbar spinal stenosis treated with OLIF surgery. These findings contribute to the growing body of evidence supporting the use of this technique in clinical practice.

## Data Availability

Not applicable.
